# Novel Frog Skin-Derived Peptide Dermaseptin-PP for Lung Cancer Treatment: *In vitro/vivo* Evaluation and Anti-tumor Mechanisms Study

**DOI:** 10.3389/fchem.2020.00476

**Published:** 2020-06-05

**Authors:** Ziyi Dong, Haiyan Hu, Xianglong Yu, Li Tan, Chengbang Ma, Xinping Xi, Lei Li, Lei Wang, Mei Zhou, Tianbao Chen, Shouying Du, Yang Lu

**Affiliations:** ^1^Laboratory of Traditional Chinese Medicine, School of Chinese Materia Medica, Beijing University of Chinese Medicine, Beijing, China; ^2^Livzon Pharmaceutical Group Inc., Zhuhai, China; ^3^Natural Drug Discovery Group, School of Pharmacy, Queen's University, Belfast, United Kingdom; ^4^Department of Biotechnology, Beijing Institute of Radiation Medicine, Beijing, China

**Keywords:** novel peptide discovery, molecular cloning, peptide synthesis, anti-lung cancer, membrane destruction, apoptosis

## Abstract

Lung cancer is the major cause of cancer deaths worldwide, and it has the highest incidence and mortality rate of any cancer among men and women in China. The first-line therapy for lung cancer treatment is platinum-based chemotherapy drugs such as cisplatin. However, the application of present chemotherapies is limited by severe side effects, which stimulates the discovery of new drugs with new anti-tumor mechanisms and fewer side effects. Beneficially, many antimicrobial peptides (AMPs) from frog skin have been reported to exhibit potent anti-cancer activities with low toxicity, high selectivity and a low propensity to induce resistance. In this study, we first reported an AMP named Dermaseptin-PP, from a rarely studied frog species, *Phyllomedusa palliata*. Dermaseptin-PP exhibited selective cytotoxicity on H157, MCF-7, PC-3, and U251 MG cancer cells instead of normal HMEC-1 cells with low hemolytic effect. Furthermore, on subcutaneous H157 tumor model of nude mice, Dermaseptin-PP was found to display potent *in vivo* anti-tumor activity in a dose-related manner without obvious hepatopulmonary side effects. It is widely accepted that AMPs usually work through a membrane disruptive mode, and the confocal laser microscope observation confirmed that Dermaseptin-PP could destroy H157 cell membranes. Further investigation of mechanisms by flow cytometry assay and immunohistochemical analysis unraveled that Dermaseptin-PP also exerted its anti-tumor activity by inducing H157 cell apoptosis via both endogenous mitochondrial apoptosis pathway and exogenous death receptor apoptosis pathway. Herein, we emphasize that the membrane disrupting and the apoptosis activation effects of Dermaseptin-PP both depend on its concentration. Overall, a novel frog skin-derived AMP, named Dermaseptin-PP, was identified for the first time. It possesses strong antimicrobial activity and effective anti-tumor activity by distinct mechanisms. This study revealed the possibility of Dermaseptin-PP for lung cancer treatment and provided a new perspective for designing novel AMP-based anti-tumor candidates with low risk of cytotoxicity.

## Introduction

Cancer has always been a leading cause of human death, which results from the uncontrolled growth and spread of abnormal cells (Gaspar et al., 2013). Lung cancer is the major cause of cancer deaths worldwide, and it is also the type of cancer with the highest incidence and mortality among men and women in China (Ferlay et al., 2010; Viktorsson et al., 2014). Non-small-cell lung cancer (NSCLC) accounts for almost 85% of all lung cancers, and most NSCLC patients are in the advanced stages of treatment (Allingham-Hawkins et al., 2010).

Platinum-based chemotherapies, such as cisplatin, has been used as the first-line therapy for advanced NSCLC (Mi et al., 2016; Yu et al., 2018). Nevertheless, the therapeutic efficacy of the existing cancer chemotherapies is limited by narrow therapeutic indexes. Cancer chemotherapies are lethal to both cancer cells and healthy cells undergoing rapid proliferation and may suppress the immune system, causing severe side effects (Arpornsuwan et al., 2014). At the same time, drug resistance in cancer and insufficient drug concentration in tumor areas also severely limit the efficacy of chemotherapy drugs. Thus, new therapeutic anti-tumor drugs with new mechanisms and fewer side effects need to be constantly searched for (Moxley and McMeekin, 2010).

It is well-known that the granular glands on frog skin can secrete lots of antimicrobial peptides (AMPs) which possess broad spectrum antimicrobial activities (Torres et al., 2017; Li et al., 2019). Encouragingly, many AMPs were also found to possess anti-cancer activities (Baxter et al., 2017), so it has become an important research area to develop AMPs as functional therapies to combat cancer (Hancock and Sahl, 2006; Riedl et al., 2011). Structurally, AMPs usually possess cationic charges and amphipathicity resulting from their α-helical structure and thus are capable of interacting with microbial membranes and cancer cell membranes that are full of anionic compounds (Deslouches and Di, 2017). Based on the Antimicrobial Peptide Database (APD) (http://aps.unmc.edu/AP/main.php), more than 250 AMPs have been reported to exhibit anti-cancer activities, and many researchers have suggested that numerous AMPs possess the ability to destroy cancer cell membranes or promote the apoptosis of cancer cells (Qiang et al., 2014; Shi et al., 2016). As a result, AMPs are increasingly coming into focus as new strategies for cancer treatment. Importantly, the frog skin secretions from the genus *Phyllomedusa* are regarded as abundant producers of AMPs, especially to produce AMPs of phylloseptin and dermaseptin families (Nicolas and Amri, 2009).

In this study, we first identified a novel AMP, named Dermaseptin-PP, from the frog skin secretion of *Phyllomedusa palliata* (*P. palliata*), a rarely studied South American frog. Dermaseptin-PP exhibited potent antimicrobial effects against Gram-positive and Gram-negative bacteria, fungi and even four drug-resistant strains, with a relatively low hemolytic effect. Significantly, we proved that the novel isolated AMP Dermaseptin-PP exerted excellent *in vitro* anti-cancer activity against four different cancer cells with the strongest anti-cancer effect on H157 cells. Furthermore, we investigated the *in vivo* anti-tumor activity of Dermaseptin-PP using subcutaneous H157 tumor model. Results strongly suggested that Dermaseptin-PP possess a potent *in vivo* anti-tumor activity via different mechanisms including destruction of cancer cell membranes and apoptosis induction of cancer cells. Therefore, we not only supplemented the understanding of AMPs belonging to the Dermaseptin family in terms of their bioactivity and working mechanisms, but also provided a new candidate for lung cancer treatment.

## Materials and Methods

### Collection of *Phyllomedusa palliata* Skin Secretions

Specimens of *P. palliata* (*n* = 5) were obtained from a commercial source. Briefly, by applying mild transdermal electrical stimulation (5 V, 50 Hz, 4 ms plus width) to the frog skin, the holocrine glands produced the skin defensive secretions. Thereafter, the skin secretions were washed from the dorsal of frogs using deionized water, and the collections were snap-frozen in liquid nitrogen, lyophilized in an Alpha 1–2 freeze-drying system (HetoSicc, Martin Christ, Germany), and kept at −20°C. Sampling of skin secretion was carried out by Mei Zhou under the guidelines of the UK Animal (Scientific Procedures) Act 1986, project license PPL 2694, issued by the Department of Health, Social Services and Public Safety, Northern Ireland. Procedures had been vetted by the IACUC of Queen's University Belfast and approved on 1 March 2011.

### “Shotgun” Cloning of the Novel Peptide Precursor-Encoding cDNA From *Phyllomedusa palliata* Skin Secretion-Derived cDNA Library

Five-mg of lyophilized *P. palliata* skin secretion was dissolved in 1 mL of lysis/binding buffer, and then the polyadenylated mRNA was isolated using magnetic oligo-dT beads in the Dynabeads® mRNA DIRECT™ Kit (BIOTECH, UK). The extracted mRNA was reverse transcribed to synthesize the first-strand cDNA, and the cDNA was then subjected to 3'-RACE procedures to obtain the full-length pre-pro-Dermaseptin-PP nucleic acid sequence according to the instructions of the BD SMART™-RACE cDNA Amplification Kit (Clontech, Palo Alto, CA, USA). In detail, the 3′-RACE reaction applied a nested universal primer (NUP, provided in the kit) and also a degenerate sense primer (S1:5′-GGCTTYCCTGAAGAAATCTC-3′, Y = C + T) designed according to an N-terminal sequence—AS/FLKKS—of the highly conserved signal peptide of neobatrachian frog skin AMP precursors (Kurabayashi and Sumida, 2009). The PCR cycling program is shown in Supplementary Table 1. RACE-PCR products were analyzed by agarose gel electrophoresis, purified by a Cycle Pure Kit (Omega Bio-Tek, USA) and cloned using a pGEM®-T Easy vector (Promega Corporation, Southampton, UK). Finally, the nucleotide sequences of selected cloned samples were sequenced by an automated ABI 3730 sequencer (Applied Biosystems, Foster City, CA, USA). Then, each nucleic acid sequence was translated into an amino acid sequence through the ExPASy Translate Tool online portal (https://www.expasy.org). The deduced mature peptide sequence was searched in the Blast Alignment Search Tool (BLAST) (https://blast.ncbi.nlm.nih.gov/Blast.cgi) to compared it with the known peptides sequences in the protein database. Consequently, alignments of similar regions of these peptides were established by Clustal Omega (https://www.ebi.ac.uk/Tools/msa/clustalo/).

### Identification and Structural Characterization of the Novel Peptide From *Phyllomedusa palliata* Skin Secretion

An aliquot of 5 mg *P. palliata* skin secretion was dissolved in 1 mL water/TFA (99.95/0.05, v/v) and clarified by centrifugation. Thereafter, the supernatant was subjected to reversed-phase HPLC using a Cecil Adept 4200 HPLC system (Amersham Biosciences, Buckinghamshire, UK) fitted with the analytical column (C-5, 250 mm × 4.6 mm, 5 μm, Phenomenex, UK), and was eluted with a gradient elution from 100% (water/TFA) (99.95/0.05, v/v) to 100% (acetonitrile/water/TFA) (80/19.95/0.05, v/v/v) in 240 min at a flow rate of 1 mL/min. The fractions were collected automatically at 1 min intervals and were continuously detected at wavelength of 214 nm. The molecular masses of peptides in each fraction were analyzed by matrix-assisted laser desorption ionization time-of-flight mass spectrometry (MALDI-TOF MS) on a linear time-of-flight Voyager DE mass spectrometer (Voyager DE, Perspective Biosystems, Foster City, CA, USA) in the positive detection mode with alpha-cyano-4-hydroxycinnamic acid (CHCA) used as a matrix. The mass spectrometer was calibrated in the range of 1–4 kDa, and the accuracy of mass determinations was ± 0.1%. In detail, a volume of 2 μL of each HPLC fraction was spotted on a MALDI plate, and was left at room temperature to dry, followed by the addition of 1 μL CHCA matrix solution onto each spot. Then, the plate was allowed to dry again by air and loaded into the MALDI-TOF mass spectrometer. The fraction that contained the peptide with the molecular mass coincident with the computed molecular masse of the predicted mature Dermaseptin-PP deduced from encoded cDNA was further injected into a HPLC column (Jupiter, C18, 5 μm, 150 mm × 4.6 mm, Phenomenex, UK) connected to an LCQ-Fleet ESI ion-trap mass spectrometer (Thermo Fisher, San Jose, CA, USA) for primary structure identification. The flow rate was set as 20 μL/min with a linear gradient from 100% (H_2_O/formic acid) (99.90/0.10, v/v/v) to 100% (H_2_O/acetonitrile/formic acid) (19.90/80.00/0.10, v/v/v) in 135 min. The mass analysis was carried out in the positive ion mode and the acquired spectral range was 500–2,000 m/z with the relative intensity greater than 50%. The parameters of electrospray ionization ion-trap mass spectrometry (ESI/MS) were as set as follows: spray voltage of + 4.5 kV, drying gas temperature of 320°C, drying gas flow of 200 μL/min, and maximum accumulation time (ion trap) of 350 ms. The first mass analysis was performed in full-scan mode, and the peptide ions with a relative intensity greater than 50% were further fragmented by collision-induced dissociation (CID), then b and y ions were generated and detected in the second mass analyses. The spectrometer employed Xcalibur software (Thermo Fisher, San Jose, CA, USA), and data was analyzed using Proteome Discoverer 1.0 (Thermo Fisher, San Jose, CA, USA).

### Solid-Phase Peptide Synthesis (SPPS) of the Novel Peptide

The novel peptide (Dermaseptin-PP) with confirmed amino acid sequence was chemically synthesized in a Tribute automated peptide synthesizer (Protein Technologies, USA) using a solid-phase Fmoc strategy. Then, the peptide-resin powder was mixed with a cleavage cocktail (25 mL/g resin) consisting of 94% TFA, 2% Thioanisole, 2% 1, 2-Ethanedithiol and 3% H_2_O (v/v/v) and the mixture was subjected to a magnetic stirrer at room temperature for 2.5 h to complete the cleavage and deprotection steps, thereafter, the crude peptide was obtained. The crude peptide replicates were purified by reversed-phase HPLC and the highly purified peptide replicates were confirmed by MALDI–TOF mass spectrometry.

### Secondary Structure Determination of Dermaseptin-PP by Circular Dichroism (CD) Analyses

The determination of the secondary structure of Dermaseptin-PP was conducted by a JASCO J-815 circular dichroism (CD) spectrometer (Jasco, Essex, UK) at 20°C. The measuring wavelength was from 190 to 260 nm at a scanning speed of 200 nm/min, and the data pitch and bandwidth were 0.5 and 1 nm, respectively. Briefly, Dermaseptin-PP was dissolved in 10 mM ammonium acetate (NH_4_Ac) or 50% 2,2,2-trifluoroethanol (TFE) to reach the final concentration of 100 μM. In addition, the percentage of the α-helix structure of Dermaseptin-PP was estimated by HNN online software (https://npsaprabi.ibcp.fr/cgi-bin/npsa_automat.pl?page=/NPSA/npsa_hnn.html).

### Antimicrobial Assays

The minimum inhibitory concentrations (MICs) and the minimum bactericidal concentrations (MBCs) of Dermaseptin-PP were examined on different bacterial strains, including the Gram-negative bacteria *Escherichia coli* (*E. coli*) (NCTC 10418), *Klebsiella pneumoniae* (*K. pneumoniae*) (ATCC 43816), *Pseudomonas aeruginosa* (*P. aeruginosa*) (ATCC 27853), the Gram-positive bacteria *Staphylococcus aureus* (*S. aureus*) (NCTC 10788), *methicillin-resistant Staphylococcus aureus* (*MRSA*) (NCTC 12493), *Enterococcus faecalis* (*E. faecalis*) (NCTC 12697), and the yeast *Candida albicans* (*C. albicans*) (NCYC 1467). To begin with, all microorganisms were cultured at 37°C overnight in Mueller Hinton Broth (MHB) to reach their log phase, and then sub-cultured and diluted to achieve a 5 × 10^5^ CFU/mL of bacterial suspension. Subsequently, a volume of 99 μL sub-cultured microorganism was incubated with 1 μL serial concentrations of peptides (from 1 to 64 μM) in 96-well plates at 37°C for 24 h. The growth of microorganisms was detected at a wavelength 550 nm using a Synergy HT plate reader (Biotech, USA). Thereafter, 10 μL of solutions in wells with no apparent growth of microorganism were spotted on Mueller agar (MHA) plates in triplicate and the plates were incubated overnight at 37°C to determine the MBC values. The MBC value was defined as the lowest concentration of peptide without microbial growth.

### Hemolysis Assays

The hemolytic effect of Dermaseptin-PP was determined using a pre-washed 4% suspension of horse red blood cells (supplied by TCS Biosciences Ltd, UK). A volume of 100 μL peptide in concentration gradients (ranging from 1 to 64 μM) was incubated with the same volume of 4% red blood cells in a 96-well plate at 37°C for 2 h. Additionally, horse red blood cells added with the same volume of 2% Triton X-100 (Sigma Aldrich, USA) or PBS were set as the positive or negative control respectively. The optical density (OD) value of each well at 550 nm was measured using a Synergy HT plate reader (Biotech, USA). The hemolytic rate was calculated by the following formula:

$$\begin{array}{l} \text{Hemolytic rate }=\frac{\text{OD Sample }-\text{ OD Negative }}{\text{OD Positive }-\text{ OD Negative}}\times 100\text{ \%} \end{array}$$

Then, the Half maximal hemolysis concentration (HC_50_) was determined using GraphPad Prism 6.0 (GraphPad Software, USA).

### Cell Line, Cell Culture, and Chemicals

Four cancer cell lines, including Human non-small cell lung cancer cell line H157 (ATCC-CRL-5802), Human breast adenocarcinoma cell line MCF-7 (ATCC-HTB-22), Human prostate carcinoma cell line PC-3 (ATCC-CRL-1435), Human neuronal glioblastoma cell line U251MG (ECACC General Cell Collection: 09063001), and one normal cell line, Human microvascular epithelial cell line HMEC-1 (ATCC-CRL-3243), were utilized to estimate the antiproliferation effects and the cytotoxicity of Dermaseptin-PP. All cancer cells were cultured with full-growth medium supplemented with 1% penicillin-streptomycin solution (Sigma, UK) and 10% fetal bovine serum (FBS) (Sigma, UK). H157 and PC-3 cells were cultured in RPMI-1640 medium (Invitrogen, Paisley, UK), MCF-7 and U251MG cells were grown in Dulbecco's Modified Eagle's medium (DMEM) (Sigma, St. Louis, MO, USA), while HMEC-1 cells were cultured in full-growth MCDB-131 medium (Gibco, Paisley, UK) with 10 ng/mL epidermal growth factor (EGF) and 10 mM L-Glutamine.

### MTT Cell Proliferation Assay and LDH Cell Membrane Alteration Evaluations Cell Line, Cell Culture, and Chemicals

The antiproliferative effects of Dermaseptin-PP on H157, MCF-7, PC-3, and U251 MG cancer cell lines were assessed by 3-(4,5-*dimethylthiazol*-2-*yl*)-2,5-diphenyltetrazolium bromide (MTT) cell viability assay. At first, the cells were plated in the 96-well plate with a density of 5.0 × 10^3^ cells/100 μL/well and cultured at 37°C under 5% CO_2_ for 24 h. The cells were then starved by serum-free medium for 6 h, followed by treatment with Dermaseptin-PP serial dilutions (10^−4^-10^−9^ M, *n* = 5) for 24 h. Additionally, cells in vehicle control group were treated with 1% PBS. After exposure for 24 h, a volume of 10 μL MTT (5 mg/mL) was added to each well and co-incubated for 4 h at 37°C under 5% CO_2_. Subsequently, the solution in each well was removed and 100 μL DMSO was added, and the OD value of each well was then measured at 490 nm by a Synergy HT plate reader (Biotech, USA). Also, the half maximal inhibitory concentration (IC_50_) values of Dermaseptin-PP were determined by GraphPad Prism 6.0 (GraphPad Software, USA). Importantly, the H157 cells, on which the testing novel peptide Dermaseptin-PP revealed to have the most significant anti-cancer activity would be selected for further mechanism investigation and *in-vivo* assays.

The membrane integrity of H157 cancer cells and normal HMEC-1 cells was measured by Lactate dehydrogenase (LDH) release assay to estimate the cytotoxicity of Dermaseptin-PP. When cells are damaged and the cell membrane permeability is increased, LDH will be released into the culture medium and can be detected. The degree of LDH release from destroyed H157 and HMEC-1 cells after Dermaseptin-PP treatment was determined using the Pierce LDH Cytotoxicity Assay Kit (Thermo Fisher Scientific, USA). In brief, five thousand cells/well in 100 μL full-growth medium were seeded into 96-well plates and cultured for about 24 h to 80% confluency. Then, the cells were treated with gradient concentrations of Dermaseptin-PP (10^−9^-10^−4^ M) or 1% PBS for another 24 h at 37°C under 5% CO_2_ as sample groups and a negative control group, respectively. In addition, the cells of the positive group were mixed with 10 μL Lysis buffer and incubated at 37°C under 5% CO_2_ for about 30 min to produce a relatively maximum LHD release. Then, 50 μL supernatant of each group was transferred to another 96-well plate in quintuplicate wells, 50 μL of reaction buffer was then added to each well and incubated for less than 30 min at room temperature. Finally, after the addition of 50 μL stop solution and the removal of bubbles in each well, the OD value of each well was detected at 490 nm using a Synergy HT plate reader (Biotech, USA). The percentage of LDH release in different groups was determined by the following formula:

$$\begin{array}{l} \text{LDH release }=\frac{\text{OD sample }-\text{ OD Vehicle }}{\text{OD Positive }-\text{ OD Vehicle}}\;\times 100\text{ \%} \end{array}$$

### Immunofluorescence Imaging of H157 Cell Membrane and Nucleus

Immunofluorescence imaging can directly reflect the destructive effect of Dermaseptin-PP on H157 cell morphology. In this assay, the cell morphology destructive effects on H157 cells after various concentrations of Dermaseptin-PP (from 10^−4^ to 10^−9^ M) treatment for 24 h or after Dermaseptin-PP (at concentration of 10^−4^ M) treatment for different times (30 min, 1, 2, 4, 8, 16, 24 h) were both evaluated. At first, about 2.0 × 10^5^ H157 cells in 2 mL full-growth DMEM medium were planted into each confocal dish culture plate and allowed to attach at 37°C for 24 h under 5% CO_2_. After that, the medium in each culture plate was replaced by different concentrations of Dermaseptin-PP (10^−4^-10^−9^ M, *n* = 3) and incubated for another 24 h, while the cells treated by Dermaseptin-PP (10^−4^ M, *n* = 3) were incubated for a different time (30 min, 1, 2, 4, 8, 16, 24 h). After incubation, the cells were washed twice by PBS, then dyed with 100 μL Dil (1:50) (Solarbio, Beijing) for 20 min at 37°C. Subsequently, the cells were washed again by PBS, and fixed with 500 μL 4% paraformaldehyde (Keygen, Nanjing) for 20 min at 4°C, then washed again by PBS and finally dyed with 100 μL DAPI (1:100) for 5 min at room temperature. The cell membrane was dyed red by Dil while the nucleus was dyed blue by DAPI. Lastly, the cells were washed twice with PBS and stored in 1 mL HBSS (Gibco, USA), and then observed under a FluoView FV1000 confocal microscope (Olympus Corporation, Japan), and analyzed by the Olympus Fluoview Ver1.7b viewer (Olympus Corporation, Japan).

### Apoptosis Detection by Flow Cytometry Assay

Flow cytometry experiments were utilized to further explore the possible mechanism of peptide-mediated death of H157 cells. H157 cells were seeded into the 6-well plate with the density of 3.0 × 10^5^ cells/well and allowed to attach for 24 h at 37°C under 5% CO_2_. Thereafter, the medium was replaced by 2 mL of various concentrations of Dermaseptin-PP (10^−4^-10^−7^ M). Besides, the blank control group was cells without Dermaseptin-PP treatment. After 24 h of treatment, the cells were washed twice with PBS and then dyed with the Annexin V-FITC/propidium iodide (PI) apoptosis detection kit (BD Biosciences, UK). In short, the peptide-treated cells were digested by 0.25% trypsin and then centrifuged at 1,000 rpm for 5 min to collect the cells which were then re-suspended in 200 μL binding buffer. Thereafter, the cells were dyed with 5 μL Annexin V-FITC for 5 min followed by dyed with 5 μL PI for 10 min, and ultimately analyzed by FACSCalibur flow cytometer (BD Biosciences, USA).

### *In-vivo* Anti-tumor Activity of Dermaseptin-PP

#### Animals

Balb-c nude mice (19–22 g, male) were purchased from the Beijing Weitong Lihua Experimental Animals Co. LTD (Beijing, China). Animal care was performed in accordance with the guidelines of the Ministry of Science and Technology of China and the relevant ethical norms of Beijing University of Chinese Medicine. The protocol of the current study was approved by the Ethical Committee (25-3-2019) for Laboratory Animals of Beijing University of Chinese Medicine (No: BUCM-4-2019032502-1072). All experimental procedures were designed to minimize animal suffering and the number of animals used.

#### Establishment of Subcutaneous H157 Tumor Model in Nude Mice

The *in-vivo* anti-solid tumor activity of Dermaseptin-PP was assessed on the subcutaneous H157 tumor model. A volume of 100 μL single-cell suspension containing 1 × 10^7^ H157 cells was subcutaneously injected into the right underarm of each mouse. The mice bearing day 14 tumors (the mean tumor volume was 200–250 mm^3^) representing advanced tumors were ready to receive medication, and were divided into five groups (*n* = 5), including a model group (PBS), a positive control group (Cisplatin, DDP 0.7 mM) and high, medium and low dose of Dermaseptin-PP groups (8, 6, and 4 mM). Besides, mice without tumor cells were used as a blank group (*n* = 5).

#### *In-vivo* Administration of Dermaseptin-PP

Mice of three Dermaseptin-PP groups received daily intratumoral injections with a 20-μL volume of Dermaseptin-PP (8, 6, 4 mM) for 10 days. In addition, mice in the model group were injected daily with 20 μL saline into the tumors for 10 days, while the mice in DDP group received 5-day intraperitoneal injections with 200 μL DDP (0.7 mM) and then normally fed until day 10. The mice weight and the tumor volume which was determined by [(length × width^2^)/2] were measured once in 2 days. Twenty-four hours after the last injection, the mice were sacrificed and the tumors were completely excised, embedded in paraffin, deparaffinized, sectioned, stained for TUNEL or stained with HE to reveal tumor histopathologic structures and the apoptotic effects of Dermaseptin-PP. The changes of different kinds of apoptosis related proteins including Apaf-1, caspase-9, caspase-3, cytochrome c, Fas, FasL and FADD, were evaluated by specific antibodies (Cell Signaling, Danvers, MA, USA) using a microscope (BX53; Olympus, Tokyo, Japan). The average optical density of each image was analyzed by Image-Pro Plus software. Besides, the spleen of each mouse was also removed and weighed by an electronic balance and the spleen index was calculated as ratio of spleen weight (g) to mouse body weight (g).

### Statistical Analysis

Each experiment was repeated three times and the results were presented as mean ± SEM determined by two-tailed Student's *t*-tests or one-way ANOVA. Data was statistically analyzed using GraphPad Prism 6.0 (GraphPad Software, USA).

## Results

### “Shotgun” Cloning of a Novel Peptide Precursor-Encoding cDNA

**Figure 1:**
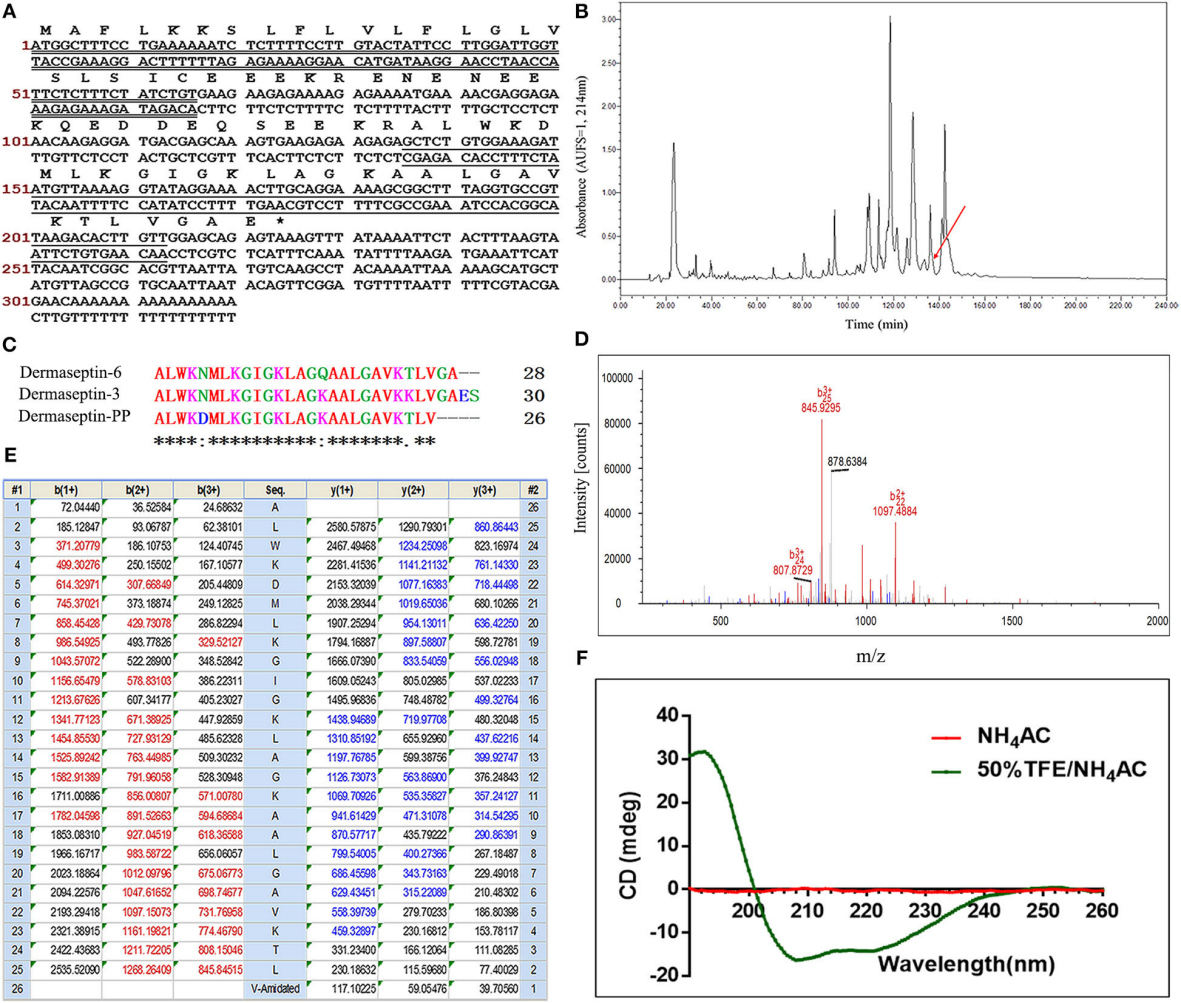
**(A)** Nucleotide sequence of cDNA cloned from *Phyllomedusa palliata* skin secretion and the corresponding translated amino acid sequence of the open reading frame of Dermaseptin-PP precursor. The putative signal peptide is double-underlined; the mature peptide is single-underlined; the termination codon is marked by an asterisk. **(B)** RP-HPLC chromatogram of *P. palliata* skin secretion at a wavelength of 214 nm with a flow rate of 1 mL/min in 240 min. The retention time (at 136.5 min) of Dermaseptin-PP is indicated by an arrow. **(C)** Alignment of cDNA deduced mature Dermaseptin-PP sequence with the top 2 non-repetitive similar dermaseptin peptide sequences from BLAST analysis. An “*” represents conserved residues; a “:” represents very similar residues and a “.” indicates similar residues. **(D)** Annotated LCQ tandem mass (MS/MS) fragmentation spectrum of Dermaseptin-PP. **(E)** Predicted single-, double-, and triple-charged b- and y-ion series arising from LCQ MS/MS fragmentation of Dermaseptin-PP. The actually observed fragment ions following actual fragmentation are severally colored red (b-ions) and blue (y-ions). **(F)** The circular dichroism (CD) spectra of pure Dermaseptin-PP in 10 mM ammonium acetate (NH_4_Ac) water solution (red line) and 50% 2,2,2-trifluoroethanol (TFE)-10 mM NH_4_Ac water solution (green line).

The full-length cDNA, encoding the biosynthetic precursor of Dermaseptin-PP, was successfully cloned from the cDNA library constructed from the skin secretion of *P. palliata*. The coding region of the cDNA sequence contains a total of 74 amino acids consisting of four domains: a putative signal peptide region located at the N-terminus of the open reading frame which is composed of 22 amino acids, a 23-residue acidic spacer domain ended with the Lys-Arg (KR) convertase processing site, a 26-residue acidic mature peptide domain with glycine-72 acting as an amide donor, and a C-terminal untranslated region (Figure 1A). The deduced mature peptide sequence was ALWKDMLKGIGKLAGKAALGAVKTLV-NH_2_.

Compared with the identified peptide sequences in the BLAST database, the mature peptide sequence showed 92% identities to both Dermaseptin-6 (accession P84926) from *Phyllomedusa tarsius* and Dermaseptin-3 (accession P80279) from *Phyllomedusa sauvagii*, suggesting that the novel peptide could be grouped under the antimicrobial peptide Dermaseptin family and it was therefore named as Dermaseptin-PP. The alignments of these three peptides were obtained via Clustal Omega analysis (Figure 1C). The Dermaseptin-PP precursor nucleotide sequence has been stored in the GenBank Nucleotide Sequence Database (https://www.ncbi.nlm.nih.gov/genbank/) with the accession number of MN17142L2.

### Primary Structure Determination and Chemical Synthesis of Dermaseptin-PP

The fractioned skin secretion of *P. palliata* by RP-HPLC was analyzed by MALDI-TOF MS to determine the molecular weight. The fraction at 136-137 min showed the identical molecular weight with Dermaseptin-PP [2652.307 (av.) Dalton (Da)] (Figure 1B). Then, the fraction at 136-137 min was subjected into the LCQ-Fleet ion-trap mass spectrometer for further primary structure identification. As shown in Figures 1D,E, the primary structure of Dermaseptin-PP was confirmed by LCQ tandem mass spectrometric (MS/MS) fragmentation. Dermaseptin-PP was demonstrably verified with the amidated C-terminal post-translational modification. Ultimately, the primary sequence of Dermaseptin-PP was unequivocally identified as ALWKDMLKGIGKLAGKAALGAVKTLV-NH_2_.

To obtain enough Dermaseptin-PP replicates, the peptide was then chemically synthesized by solid-phase Fmoc chemical strategy using a Tribute automated peptide synthesizer (Protein Technologies, USA). Thereafter, the crude synthetic peptide replicates were purified by RP-HPLC (Supplementary Figure 1) and the purified products were subjected to MALDI-TOF MS to authenticate the molecular mass (Supplementary Figure 2).

### Secondary Structure Determination of Dermaseptin-PP by Circular Dichroism (CD)

In the CD spectra (Figure 1F), Dermaseptin-PP was proved to possess a typical α-helical structure in 50% TFE-10 mmol/L NH_4_Ac solution (membrane-mimic hydrophobic environment) with the presence of a positive band at about 193 nm and the double-negative dichroic bands at around 208 and 222 nm, and the calculated α-helicity was 88%. However, Dermaseptin-PP showed almost no α-helical structure in 10 mmol/L NH_4_Ac solution (mimicking aqueous environment). The results suggested that Dermaseptin-PP could serve as an amphipathic α-helical structure under membrane-mimic environments so that it could exhibit the strong antimicrobial and anti-tumor effect via a membrane-disrupting action.

### Antimicrobial and Hemolytic Activities of Dermaseptin-PP

**Figure 2:**
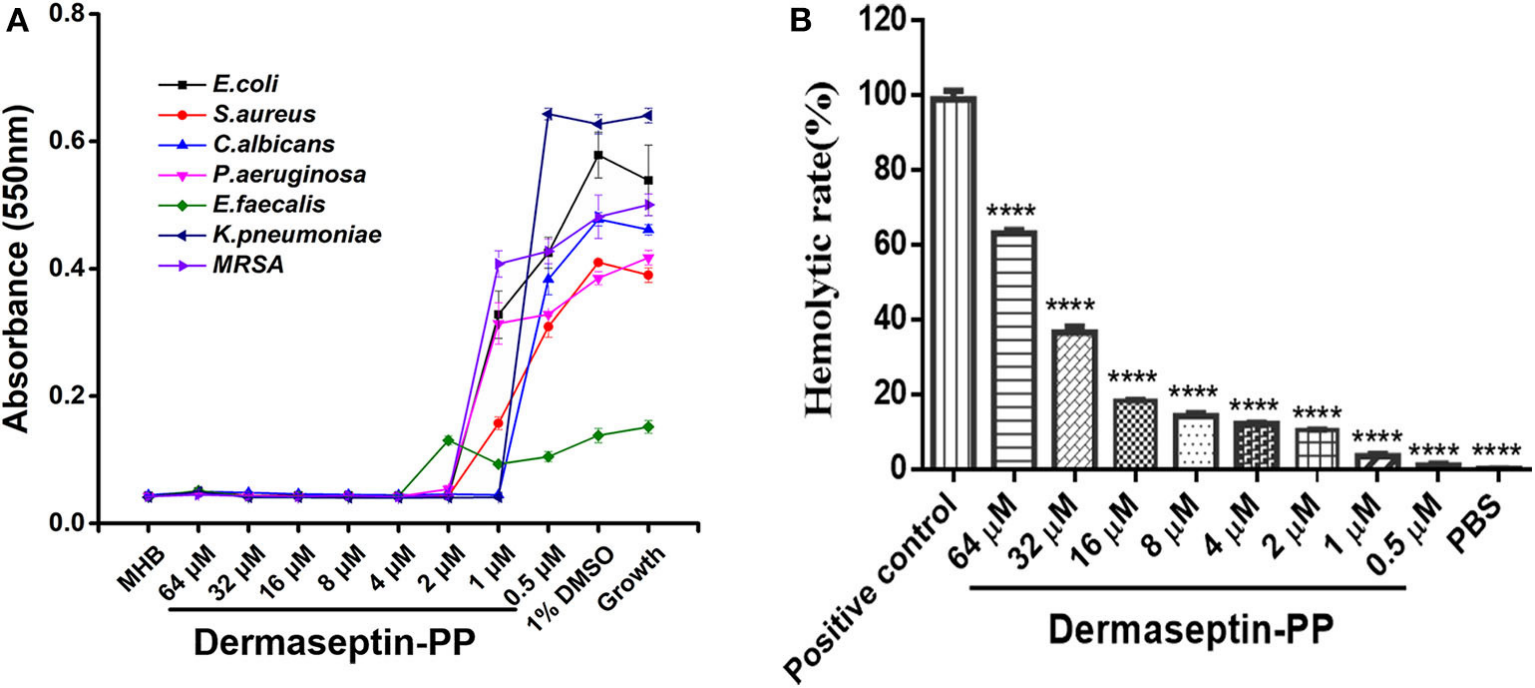
**(A)** The antimicrobial activity of Dermaseptin-PP against the growth of *E. coli, S. aureus, C. albicans, P. aeruginosa, E. faecalis, K. pneumoniae*, and *MRSA*. The data represents means ± SEM (standard error of the mean) of three independent experiments. MHB is blank control; 1% DMSO is vehicle control; Growth represents the growth control (normally growing microorganism in MHB). The Y-axis represents absorbance, higher absorbance refers to more visible microorganisms, indicating lower antimicrobial activity. **(B)** The hemolytic activity of Dermaseptin-PP (at concentration from 0.5 to 64 μM) on horse erythrocytes. Values are the mean ± SEM for three independent experiments. The significance of group difference was determined by Dunnett's multiple comparisons of one-way ANOVA in GraphPad Prism (version 6.0) software. ****P < 0.0001 vs. positive control (2% Triton X-100).

The antimicrobial activity of Dermaseptin-PP was assessed on three common strains (*E. coli, S. aureus*, and *C. albicans*) and four drug-resistance strains (*E. faecalis, P. aeruginosa, K. pneumonia*, and *MRSA*). The MIC value was defined as the lowest concentration of Dermaseptin-PP that prevented the visible growth of the microbe. Results indicated that Dermaseptin-PP exhibited strong antimicrobial activity against all strains (Figure 2A). The MIC and MBC values were summarized in Supplementary Table 2. Along with its strong antimicrobial activity, Dermaseptin-PP exhibited only a moderate hemolytic effect (<20% at 16 μM with a calculated HC_50_ of 38.77 μM) (Figure 2B) which was acceptable at its effective antimicrobial concentrations ranging from 1 to 4 μM.

### Antiproliferative Effect and Membrane-Disrupting Effect of Dermaseptin-PP on Typical Cells

**Figure 3:**
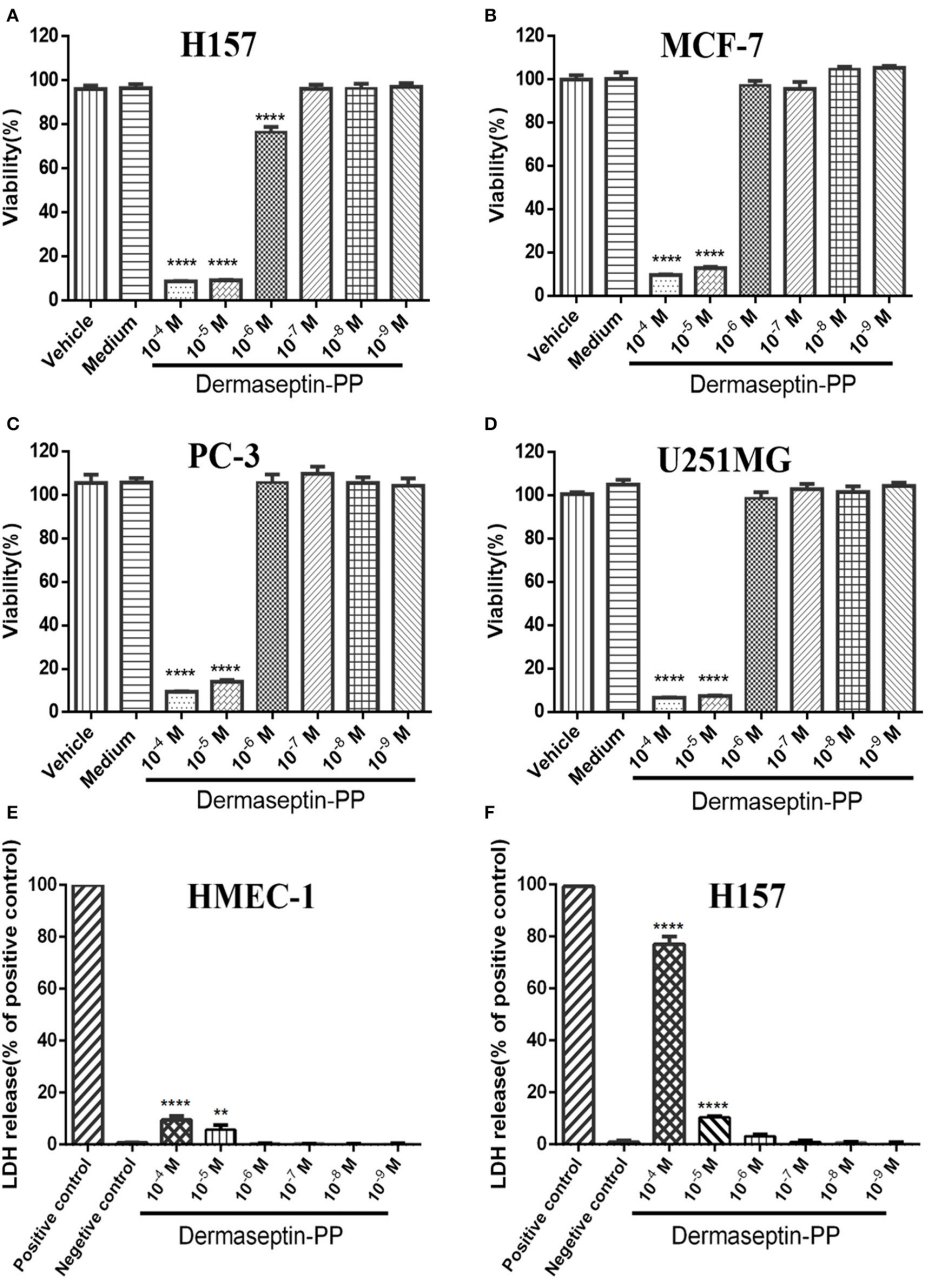
The viabilities of **(A)** H157; **(B)** MCF-7; **(C)** PC-3; **(D)** U251MG cells treated with Dermaseptin-PP at gradient concentrations from 10^−4^ to 10^−9^ M determined by MTT assays. Vehicle control: cells treated with 1% PBS; Medium control: cells treated with pre-warmed serum-free medium. Values are mean ± SEM of three independent experiments. Data was analyzed with Dunnett's multiple comparisons of one-way ANOVA using GraphPad Prism. The significance was expressed as *p*-values: ****P < 0.0001 vs. vehicle control. The percentage of LDH release from **(E)** HMEC-1 cells and **(F)** H157 cells treated by Dermaseptin-PP (10^−4^-10^−9^ M). Values are mean ± SEM of three independent experiments. Data was analyzed with Dunnett's multiple comparisons of one-way ANOVA using GraphPad Prism. “****” indicates *p* < 0.0001 and “**” represents *p* < 0.01 compared with negative control (1% PBS).

Because of the specific characteristics (being cationic and amphipathic), many AMPs have been reported to possess anti-cancer activities. In this study, Dermaseptin-PP was found to possess a significant antiproliferative effect on all tested cancer cell lines, especially on H157 cells (Figures 3A–D), at concentrations of 10^−4^ and 10^−5^ M. The calculated half maximal inhibitory concentrations (IC_50_) of Dermaseptin-PP on H157, MCF-7, PC-3, and U251 MG cells were 1.55, 2.92, 4.15, and 2.47 μM. It was noteworthy that all the IC_50_ values were much lower than the HC_50_ value (38.77 μM), indicating that Dermaseptin-PP was safe to be used as an anti-cancer agent. In addition, the LDH release examinations indicated that Dermaseptin-PP induced a very low degree of LDH release in normal HMEC-1 cells (<10% at 10^−4^ M) (Figure 3E) while Dermaseptin-PP (at 10^−4^ M) induced nearly 80% LDH release in H157 cancer cells (Figure 3F), suggesting that Dermaseptin-PP had almost no cytotoxicity on normal cells, but showed strong membrane-disrupting effect on H157 cancer cells.

### Dermaseptin-PP Induced H157 Cell Morphological Changes

**Figure 4:**
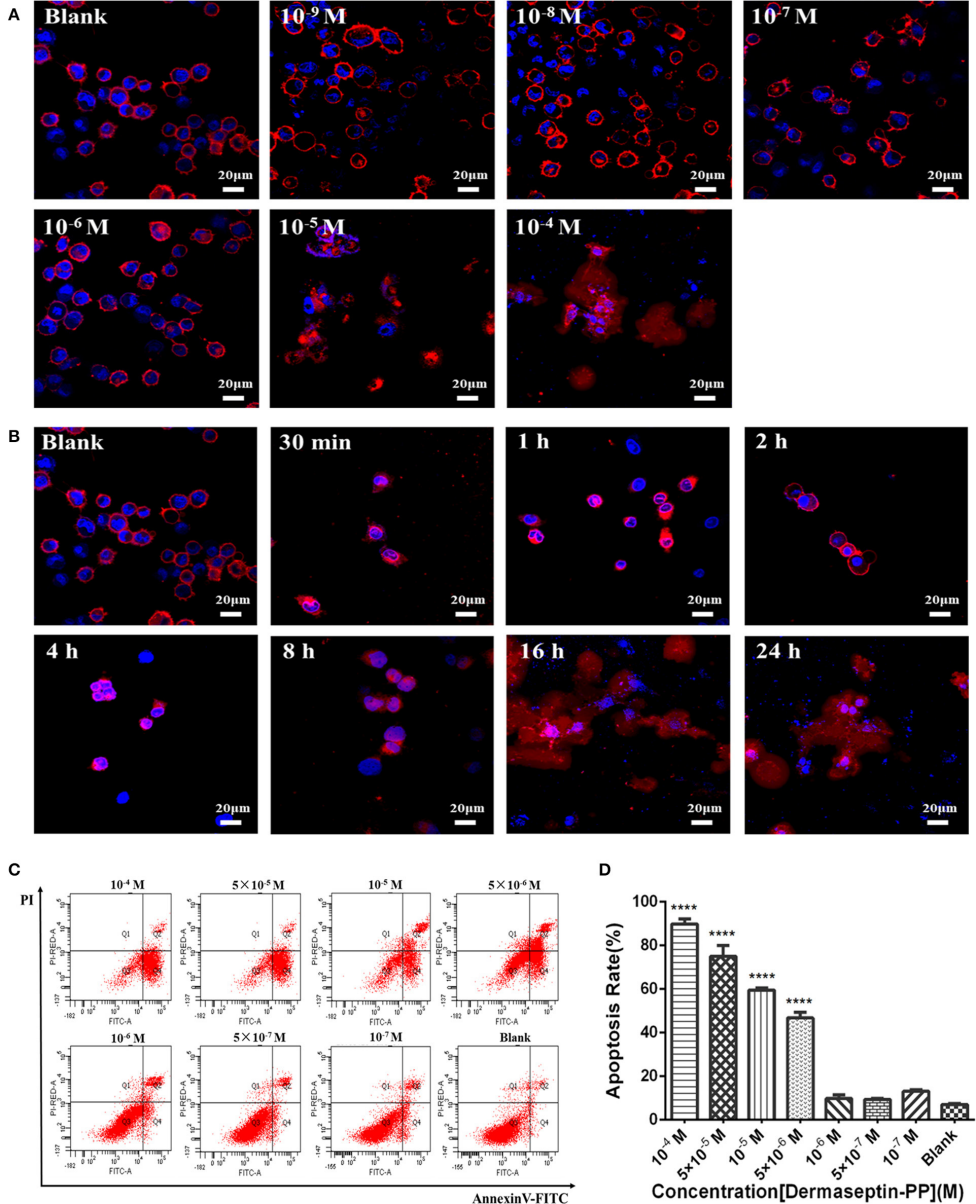
**(A)** The morphology changes of H157 cells after treatment with gradient Dermaseptin-PP (10^−4^ to 10^−9^ M) for 24 h. **(B)** The morphology changes of H157 cells after Dermaseptin-PP (10^−4^ M) treatment for different time (30 min, 1, 2, 4, 8, 16, 24 h). The cell membrane and the nucleus were dyed red and blue respectively. The images were observed under the laser confocal microscope by a magnification factor × 60. **(C)** The representative images of H157 cells treated with different concentrations of Dermaseptin-PP from flow cytometry analysis. **(D)** The apoptosis rate of H157 cells treated by gradient Dermaseptin-PP (10^−4^ to 10^−9^ M) was quantified based on flow cytometry analysis. Dermaseptin-PP could promote apoptosis of H157 cells from concentration of 5 × 10^−6^ M. All data was represented as the mean ± SEM of three independent experiments. The significance of group differences was determined by *P*-value. “****” represents *P* < 0.0001 vs. blank control group (cells without Dermaseptin-PP treatment).

As Dermaseptin-PP is a cationic AMP, it can interact with the anionic phospholipid composition of cell membranes due to the initial electrostatic attraction, and then its amphipathic formation will be induced to ensure its insertion into the cell membrane. Thus, Dermaseptin-PP was speculated to possess the membrane-disrupting effect, which was then proved by its role in promoting H157 cell morphological changes. In this assay, the cell membrane was dyed red by Dil, and the nucleus was dyed blue by DAPI, so the cell morphological changes could be observed under the laser confocal microscope. Results showed that after 24 h treatment, Dermaseptin-PP at low concentrations (10^−9^-10^−6^ M) showed negligible destructive effects on both H157 cell membranes and nuclei while Dermaseptin-PP at concentrations of 10^−5^ M and above exerted a severely destructive effect on H157 cell membranes (Figure 4A), suggesting that the Dermaseptin-PP could indeed serve a dose-related membrane-disrupting action. Furthermore, we also observed how the membrane-disrupting effect of Dermaseptin-PP (at 10^−4^ M) changed over time. Interestingly, as time went on, the cell membrane began to rupture and dispersed at the 8th hour and the destruction degree were increasingly severe until the 24th hour, revealing a time-dependent membrane-disrupting effect of Dermaseptin-PP (Figure 4B).

### Dermaseptin-PP Induced H157 Cell Death via Induction of Apoptosis at the Cellular Level

Apart from a non-specific interaction with eukaryotic cell membranes, the anti-tumor mechanism of the cationic AMPs may also be the result of internalization apoptosis induction of cancer cells. In this part, H157 cells were co-incubated with different concentrations of Dermaseptin-PP ranging from 10^−7^ to 10^−4^ M for 24 h, then stained by Annexin V/PI apoptosis kit and detected by flow cytometer. The image from flow cytometry analysis divided the cells into four groups: dead cells (Annexin V–/PI+), early apoptotic cells (Annexin V+/PI–), normal living cells (Annexin V–/PI–) and late apoptotic cells (Annexin V+/PI+) (Figure 4C). Moreover, flow cytometry analysis indicated that the apoptosis rate in H157 cells significantly increased in Dermaseptin-PP groups compared with the blank control group, reaching to 90% apoptosis in 10^−4^ M Dermaseptin-PP group. It was noteworthy that the higher the concentration of Dermaseptin-PP, the higher the apoptosis rate was induced, showing a dose-dependent manner (Figure 4D).

### Dermaseptin-PP Inhibited the Growth of Subcutaneous H157 Tumor in Nude Mice Without Obvious Side Effects

**Figure 5:**
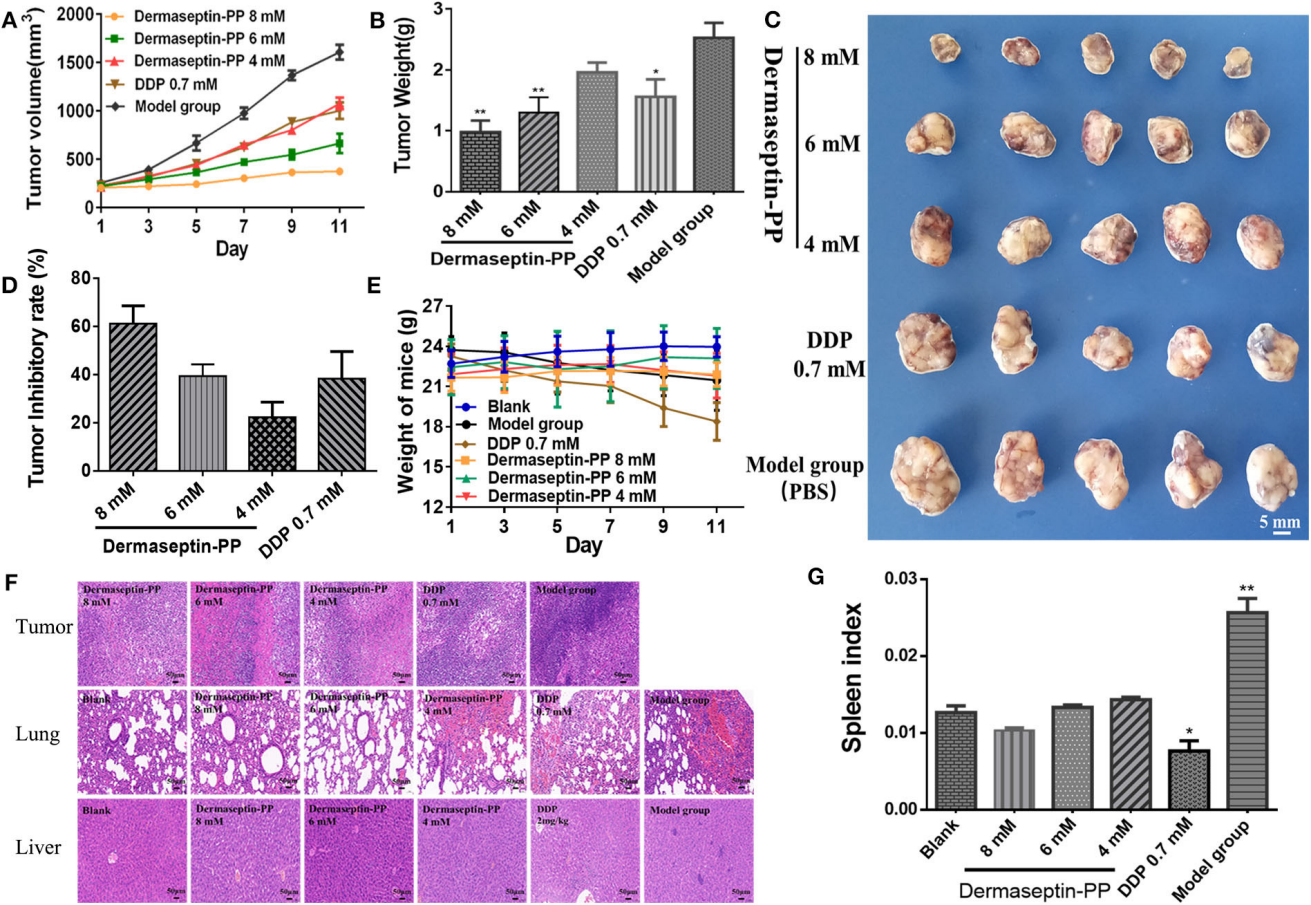
**(A)** Tumor volume of all administration groups measured every 2 days with SEMs shown as error bars around the mean of each data point. **(B)** The quantification tumor weight of the excised tumor of all administration groups. Data was analyzed with unpaired Student's *t*-test using GraphPad Prism 5 software. **P < 0.01, *P < 0.05 vs. model group (PBS). **(C)** The images of the excised tumor of all administration groups. **(D)** Tumor inhibitory rate of Dermaseptin-PP and DDP. The data represent means ± SEM (standard error of the mean). **(E)** Every 2-day weight changes of mice in each group during the experiment with SEMs shown as error bars around the mean of each data point. **(F)** Representative images of HE staining (×200) of isolated tumor, lung and liver of different administration groups to evaluate the tissue histological conditions. **(G)** The calculated spleen index of mice in different groups. Data was analyzed with unpaired Student's *t*-test using GraphPad Prism 5 software. **P < 0.01, *P < 0.05 vs. blank control group.

To examine whether Dermaseptin-PP could treat established solid tumors, the inhibitory efficacy of intratumoral administration of Dermaseptin-PP for 10 consecutive days on the growth of H157 subcutaneous tumor in Balb-c nude mice was evaluated. As shown in Figure 5A, during the 10-day administration period, the tumor growth rates of mice in low dose (4 mM) and medium dose (6 mM) Dermaseptin-PP groups as well as in the positive control group (0.7 mM DDP) were strikingly reduced in comparison with the model group (PBS). Surprisingly, the tumor growth almost halted in the high dose (8 mM) Dermaseptin-PP group with no obvious tumor volume enlarge. Additionally, the quantification results of isolated tumor weights and the images of the isolated tumors were shown in Figures 5B,C, the tumor weights in the 6 and 8 mM Dermaseptin-PP groups were much lower than that in positive control group (0.7 mM DDP), indicating that Dermaseptin-PP had strong anti-tumor activity *in vivo*. Additionally, tumor inhibitory rate was estimated by the equation:

$$\begin{array}{l} \text{Tumor inhibitory rate }=\\ \frac{\text{Tumor weight }\left(\text{Model group}\right)-\text{Tumor weight }\left(\text{sample group}\right)}{\text{Tumor weight }\left(\text{Model group}\right)}\times 100\text{ \%} \end{array}$$

As expected, the tumor inhibitory rates in 6 and 8 mM Dermaseptin-PP groups were much higher compared to the other administration groups, which were 60.53 and 46.57%, respectively (Figure 5D). Results indicated that Dermaseptin-PP exerted a certain inhibitory effect on the solid tumor formed by H157 cells in nude mice, and the inhibitory effect increased with the increase of the drug dose, suggesting a dose-related action.

In addition, we also observed the survival condition, measured bodyweight changes and calculated the spleen index of all the mice. In the last few days of this experiment, mice in the model group started to eat and drink less and their skin began to lose its luster, showing a poor mental state. On the contrary, after Dermaseptin-PP treatment (especially 6 and 8 mM), the mice increased activity, drank and ate normally and showed a shiny skin and better mental state. As shown in Figure 5E, during the experiment, the mice in the Dermaseptin-PP administration groups showed a slight increase in weight, which was similar to that of the blank control group (mice without tumors). However, it was notable that the mice in the positive control group (0.7 mM DDP) showed severe weight loss and dull skin which was even worse than the model control group, reflecting the side effects of DDP. Furthermore, the spleen is an important central immune organ, and the spleen index can partly reflect the immune ability of the body. As shown in Figure 5G, compared with the blank group, the spleen index of mice in all Dermaseptin-PP groups were relatively normal like that of mice in the blank group, however, the spleen index of mice in the positive control group (0.7 mM DDP) significantly decreased. Results reflected that the chemotherapy drug, DDP, showed a subversive effect on the immune system while Dermaseptin-PP could, to some extent, protect and restore the immune system. However, the spleen index of mice in the model group was the highest, the reason maybe that pathologically, the spleen will compensatively enlarge due to the retention of immune cells in the spleen (Justo et al., 2003). Additionally, HE staining (Figure 5F) observed under the microscope revealed that in the model group, the tumor cells were densely packed and obviously heteromorphic, with severe patellar necrosis, nuclear pyknotic apoptosis of necrotic tumor and numerous neutrophils infiltrated around the necrotic lesion. Better than the model group, cancer cell growth in Dermaseptin-PP groups was inhibited, and the cell arrangement was relatively loose, cell heteromorphism and pathological mitosis were alleviated with the loss of visible tumor parenchyma. At the same time, compared with the blank group (mice without tumors), HE staining of lung and liver sections in the administration groups only resulted in the observation of a small number of neutrophils being present with no obvious tumor metastasis or other abnormalities. HE analysis gave more evidence supporting the effective *in vivo* anti-solid tumor activity of Dermaseptin-PP with few harmful side effects.

### Dermaseptin-PP Induced H157 Cell Apoptosis Through an Endogenous Mitochondrial Death Pathway and an Exogenous Death Receptor Pathway

**Figure 6:**
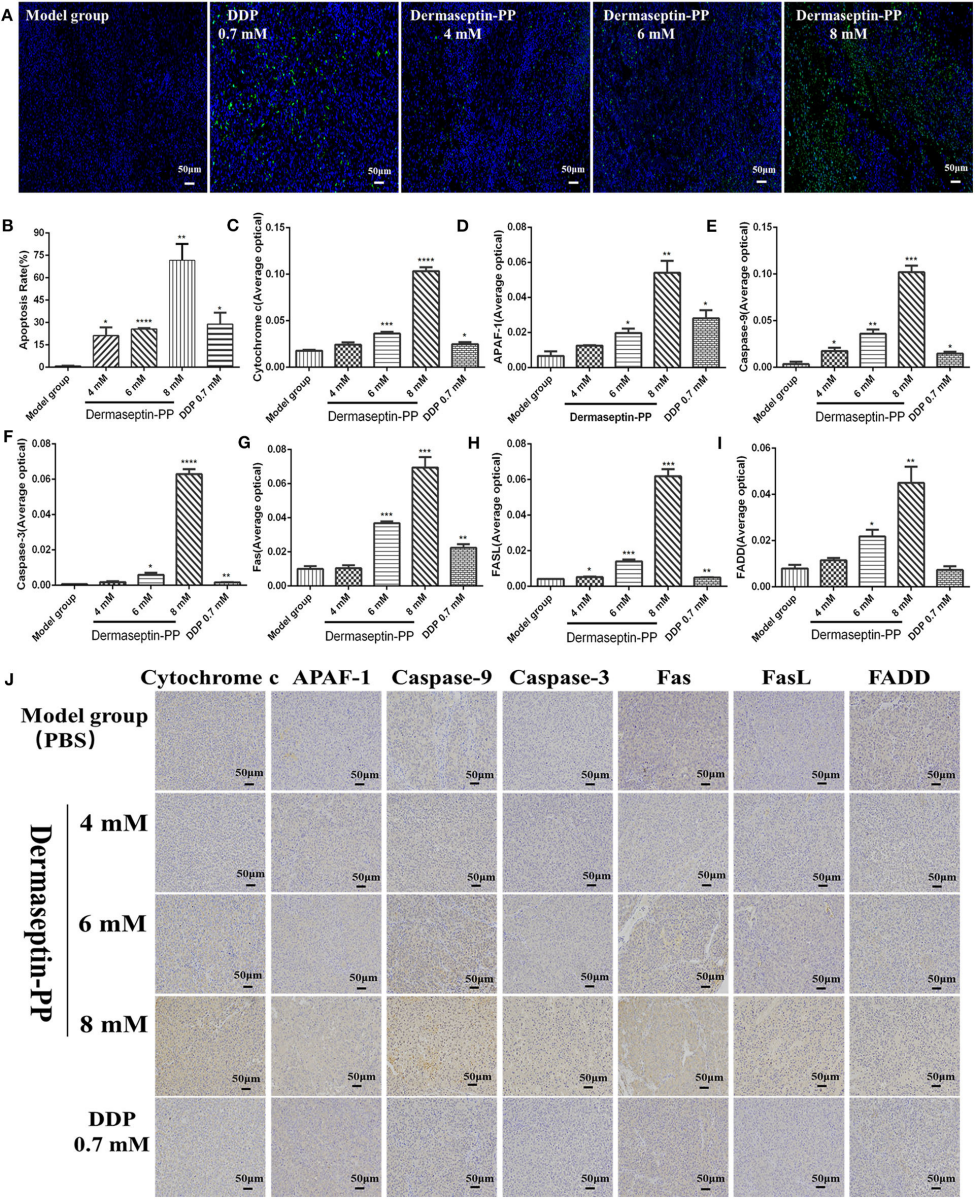
Representative images **(A)** and histological images analysis **(B)** of TUNEL staining for detection of apoptosis in H157 cells (×200). The yellow-green stained cells in the nucleus are positive (apoptotic) cells and the blue-stained cells in the nucleus are normal cells. Data was analyzed with unpaired Student's *t*-test using GraphPad Prism 5 software. ****P < 0.0001, **P < 0.01, *P < 0.05 vs. model control group (PBS). The immunohistochemical analysis was used to determine the expression of **(C)** Cytochrome c **(D)** Apaf-1 **(E)** Caspase-9 **(F)** Caspase-3 **(G)** Fas **(H)** FasL **(I)** FADD in tumor tissue isolated from mice treated under various conditions. ****P < 0.0001, ***P < 0.001, **P < 0.01, *P < 0.05 vs. model control group (PBS). **(J)** The representative images of immunohistochemical. Original magnification, ×200. The deeper the yellow, the higher the apoptosis-related protein expression. The average optical density (AOD) of each tumor tissue section was analyzed by Image-Pro Plus software and is shown in the bar graph on the right of the images. The higher the AOD, the higher the level of the apoptosis protein. Data was analyzed with unpaired Student's *t*-test using GraphPad Prism 5 software. **P < 0.01, *P < 0.05 vs. model control group.

To detect the apoptosis induction effect of Dermaseptin-PP at the level of solid tumors in mice, TUNEL (TdT-mediated dUTP Nick-End Labeling) technology was applying on the isolated tumor sections. When cells undergo apoptosis, some endonuclease enzymes will be activated to cleave genomic DNA and then expose the 3'-OH Terminal (Huang and Lu, 2001). The principle of TUNEL technology is that under the catalysis of terminal deoxynucleotidyl transferase (TdT), fluorescein-dUTP labeled with fluorescein (FITC) will be labeled on those 3'-OH terminals, so the apoptotic cells can be detected. DAPI is a common nuclear fluorescent dye which penetrates the cell membrane and combines with double-stranded DNA to produce blue fluorescence. Therefore, yellow-green stained cells in the nucleus are apoptotic cells while normal cells are only stained blue by DAPI (Figure 6A). The stained tumor sections were scanned using a Biopsy scanner (Pannoramic MIDI, 3D HISTECH). The apoptosis rate was expressed as the percentage of positive cells analyzed with 3D HISTECH Quant center Image-Pro Plus software. The results suggested that compared with the model group, the apoptosis rate of H157 cells of tumor sections treated with low, medium, and high dose Dermaseptin-PP groups (4, 6, 8 mM) and the positive drug group (0.7 mM DDP) all increased, reaching 21.21, 25.64, 71.65, and 28.83%, respectively (Figure 6B). The results were consistent with that in the flow cytometry assay.

To further explore the pathway of Dermaseptin-PP-induced apoptosis, the expression of apoptosis- regulated proteins (Apaf-1, caspase-9, caspase-3, cytochrome c, Fas, FasL, and FADD) were detected by immunohistochemical staining. Cell apoptosis mainly consists of two pathways: an endogenous mitochondrial death pathway and an exogenous death receptor pathway (Repický et al., 2008), and the selected seven apoptosis-regulated proteins are the key proteins in the two apoptosis pathways.

The representative immunohistochemical staining images were presented in Figure 6J, the quantified immunohistochemical results showed that Dermaseptin-PP concentration-dependently increased the expression of cytochrome c, Apaf-1, caspase-9, and caspase-3 in the mitochondrial pathway (Figures 6C–F), and also increased the expression of Fas, FasL and FADD in the death receptor pathway (Figures 6G–I). The above results indicated that Dermaseptin-PP could induce H157 cell apoptosis via both endogenous mitochondrial pathway and exogenous death receptor pathway.

## Discussion

In recent years, the emergence of drug-resistant cancer cells and the increased side effects of chemotherapies have prompted researchers to develop new anti-tumor agents. AMPs isolated from amphibian skin secretions are considered to be promising anti-tumor drug candidates as many of them offer a broad range of anti-cancer activities, with low toxicity to normal cells, high selectivity and a low propensity to develop resistance (Kang et al., 2012; Deng et al., 2016). Admittedly, AMPs are usually amphiphilic and positively charged (Hoskin and Ramamoorthy, 2008; Gomes et al., 2010), enable binding of AMPS to cancer cell membrane (Schweizer, 2009). Different from mammalian cell membranes which mainly consist of zwitterionic phospholipids, cancer cell membranes usually carry a higher than normal expression of negatively charged components (Giuliani et al., 2007), such as glycosaminoglycans, phosphatidylserine and negative glycoproteins (Chu et al., 2015; Yibing et al., 2015), making cancer cell membranes overall negatively charged like bacterial membranes (Theis and Stahl, 2004). Thus, the cationic and amphipathic AMPs could interact with cancer cell membranes due to electrostatic interactions and then invade cancer cells or disturb their membranes (Kang et al., 2012; Deslouches and Di, 2017). It is well-known that the genus *Phyllomedusa* is an abundant source of AMPs (Leite et al., 2008). In this research, we discovered and characterized a novel peptide, Dermaseptin-PP, from a rarely studied frog species, *P. palliata*. Also, we studied the antimicrobial activity of Dermaseptin-PP and, emphatically investigated the *in vivo* and *in vitro* anti-tumor activities and the potential anti-lung cancer mechanisms of it.

Our results confirmed that Dermaseptin-PP performed broad-spectrum antimicrobial activity at the concentrations of 1 or 2 μM (Figure 2A) with a relatively low hemolytic effect (calculated HC_50_ = 38.77 μM) (Figure 2B). Moreover, Dermaseptin-PP exhibited outstanding anti-cancer activity on all tested cancer cells (H157, MCF-7, PC-3, and U251 MG cells), which was supported by the MTT results in Figures 3A–D. It is noteworthy that, Dermaseptin-PP exerted an effective *in vivo* anti-lung-cancer effect which was mainly reflected in the reduction of tumor size and tumor weight (Figures 5A–C) while it showed no side effects on the lungs and livers of the mice (Figure 5F). To help investigate the anti-tumor mechanisms, circular dichroism analysis revealed that the cationic Dermaseptin-PP could form the amphipathic a-helical structure in a cell membrane simulation environment (Figure 1F), which helped it selectively bind with the negatively charged cancer cell membrane and then insert the hydrophobic residues into the membrane and eventually cause cell membrane rupture to exert its anti-tumor effect. Another important result was that Dermaseptin-PP (at 10^−4^ M) induced nearly 80% LDH release in H157 cancer cells (Figure 3F). LDH is a stable intracytoplasmic enzyme, which will be released into the culture medium only if the cancer cell membranes are damaged. Thus, the LDH release assay preliminary indicated that Dermaseptin-PP could destroy H157 cell membranes. Furthermore, as shown in Figure 4A, laser confocal observation confirmed that Dermaseptin-PP performed a non-specific cell membrane disruption mechanism at a concentration of 10^−5^ M and above, and this membrane-disrupting action could be further confirmed by more reliable methods such as scanning electron microscope or transmission electron microscope detection (Krysko et al., 2008; Wang et al., 2013).

Although it was widely accepted that AMPs usually act via a charge-triggered membrane destructive mode, there were also plentiful evidence supporting that AMPs could induce the activation of cell apoptosis (Jing et al., 2011; Sammy et al., 2011; Chomdao et al., 2012; Buri et al., 2013; Ting et al., 2014). The endogenous mitochondrial pathway and the exogenous death receptor pathway are the two main apoptotic pathways (Mackenzie and Clark, 2008). In the endogenous mitochondrial pathway of apoptosis, cell apoptosis is initiated by mitochondrial dysfunction and mitochondrial membrane destruction (Mignotte and Vayssiere, 1998), leading to the release of the cytochrome c (Cytc). The Cytc-induced apoptosis is mainly caused by the apoptotic protease (Caspase) pathway. After releasing into the cytoplasm, Cytc will firstly bind to the apoptotic protein actire factor (apaf-1), and then polymerize into a poly complex (Hengartner, 2000; Baksh et al., 2005). Subsequently, the fully aggregated poly complex can revitalize pro-caspases-9 and promote Cytc, dATP, apaf-1, and pro-caspase-9 to aggrege and then form apoptosome which can further activate Pro-caspases-9. Then, the downstream caspases (caspases-3, caspases-7) will be activated and finally lead to the cell death (Tang et al., 2010; Dave et al., 2011).

In the death receptor pathway, various external factors act as promoters of cell apoptosis, and then transmit apoptotic signals through different signal transduction systems, causing cell apoptosis. Fas and Fas ligand (FasL /CD95L) are considered to be the most important apoptosis-inducing molecules in various cancer cells (Fukuzawa et al., 2001; Krammer et al., 2007; Shimoyama et al., 2015). Firstly, FasL induces the trimerization of Fas and then the trimerized Fas combined with FasL, attracting Fas-associated with death domain protein (FADD) in the cytoplasm and then forming an apoptosis-inducing complex. Thus, caspase-8 will be activated, which can further activate the downstream caspases (caspase-1/3/7), resulting in the subsequent cell apoptosis (Kischkel et al., 1995; Alexander et al., 2005). In this research, using Human non-small cell lung cancer H157 cells, we examined the apoptosis activation effect of Dermaseptin-PP. Flow cytometry (Figures 4C,D) and TUNEL staining (Figures 6A,B) both suggested that Dermaseptin-PP could induce H157 cell apoptosis *in vitro* and *in vivo*. It must be noticed that, in Figures 6C–I, the immunohistochemistry data suggested that Dermaseptin-PP could significantly up-regulate the expression of the apoptosis key proteins, including cytochrome c, Apaf-1, caspase-9, and caspase-3 in the endogenous mitochondrial pathway, and Fas, FasL, and FADD in the exogenous death receptor pathway. As a consequence, we confirmed that both endogenous and exogenous apoptosis pathways were involved in Dermaseptin-PP induced H157 cell apoptosis.

## Conclusion

In summary, the first identified cationic peptide, Dermaseptin-PP, exhibited a remarkably broad spectrum of antimicrobial activity with low hemolytic cytotoxicity. More importantly, Dermaseptin-PP possessed excellent *in vitro* and *in vivo* anti-lung-cancer activity via both membrane disruption action and apoptosis activation actions. Thus, our research not only provided a hopeful candidate for the treatment of NSCLC, but also investigated the preliminary mechanism of Dermaseptin- PP induced cell apoptosis. Moreover, we found that the effects of Dermaseptin- PP are often dose-dependent, so it is important to precisely control the drug dosage to ultimately achieve a good therapeutic effect in lung cancer treatment.

## Data Availability Statement

The raw data supporting the conclusions of this article will be made available by the authors, without undue reservation, to any qualified researcher.

## Ethics Statement

The frog study was reviewed and approved by the IACUC of Queen's University Belfast, and was carried out in compliance with the guidelines of the UK Animal (Scientific Procedures) Act 1986 and project license PPL 2694, issued by the Department of Health, Social Services and Public Safety, Northern Ireland. The mouse study was reviewed and approved by the Ethical Committee (25-3-2019) for Laboratory Animals of the Beijing University of Chinese Medicine (No: 213 BUCM-4-2019032502-1072).

## Author Contributions

ZD, HH, SD, YL, TC, and LW: Conception and design. ZD, XY, HH, LT, XX, CM, and MZ: Development and performance of methodology. ZD, HH, XX, and CM: Acquisition and analysis of data. ZD, HH, and LL: Writing, review, and/or revision of the manuscript. LL, LW, MZ, SD, and YL: Supervision.

## Conflict of Interest

LT was employed by the company Livzon Pharmaceutical Group Inc. The remaining authors declare that the research was conducted in the absence of any commercial or financial relationships that could be construed as a potential conflict of interest.
