# Supplementary material for: Novel Frog Skin-Derived Peptide Dermaseptin-PP for Lung Cancer Treatment: In vitro/vivo Evaluation and Anti-tumor Mechanisms Study
Source: Front Chem. 2020 Jun 5;8:476. doi: 10.3389/fchem.2020.00476 (PMC7291860; doi:10.3389/fchem.2020.00476)
Supplement: Supplementary file 1 [file Data_Sheet_1.DOCX]

Supplementary Material

# Supplementary Figures

**
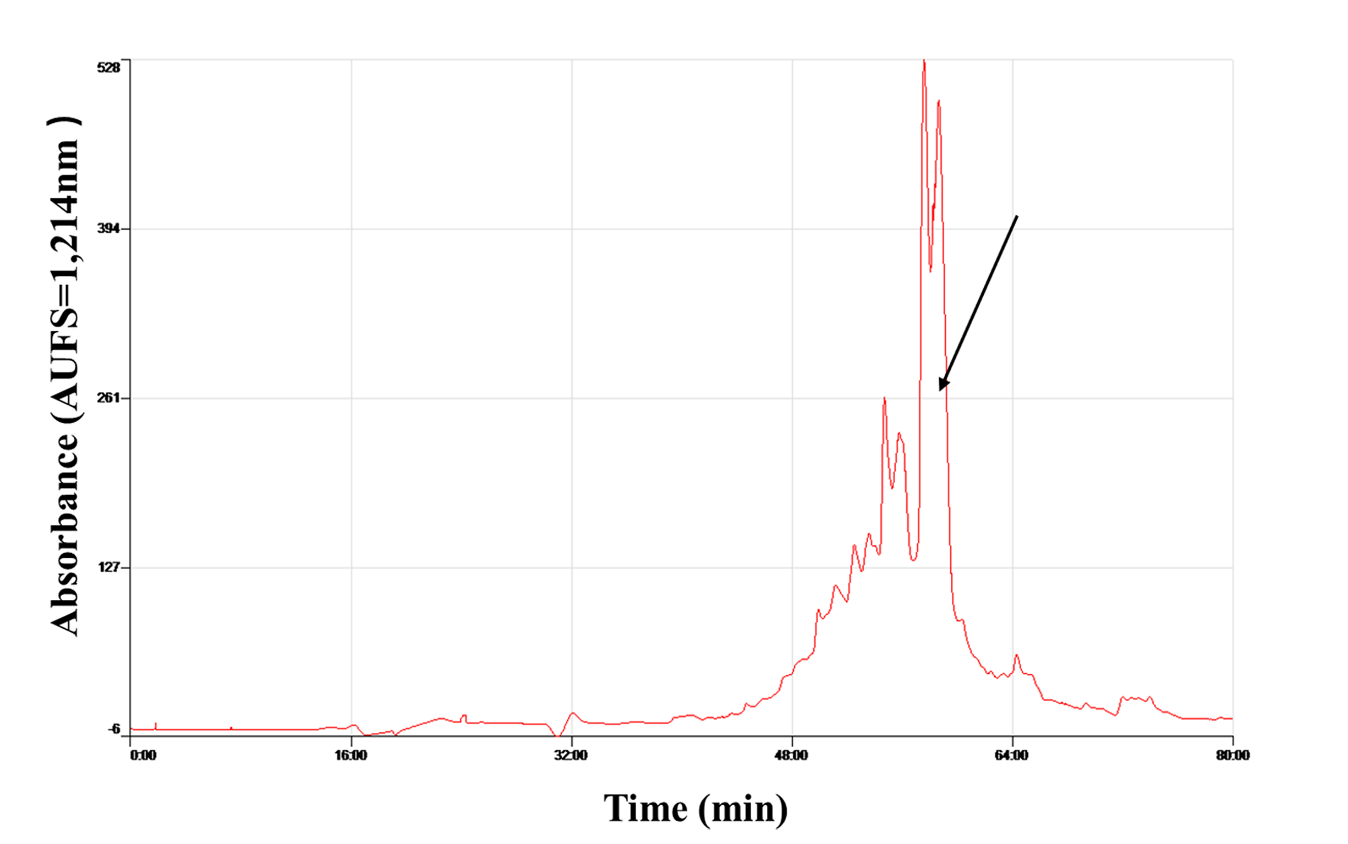
**

**Supplementary Figure 1** RP-HPLC chromatogram of the synthetic peptide Dermaseptin-PP. Dermaseptin-PP was dissolved in water/TFA (99.95/0.05, v/v) and was eluted with a gradient elution formed from 100% (water/TFA) (99.95/0.05, v/v) to 100% (acetonitrile/water/TFA) (80/19.95/0.05,119 v/v/v) in 80 min at a flow rate of 1 mL/min. The UV monitoring wavelength was 214 nm. The peak indicated by the arrow is the target peptide, Dermaseptin-PP.

**Supplementary Figure 2** MALDI-TOF spectrum of the pure peptide Dermaseptin-PP obtained from HPLC. [M+H]^+^ represents the protonated Dermaseptin-PP; [M+K]^+^ represents the Dermaseptin-PP with a potassium ion. The peak with the highest intensity (%) showed a m/z value of 2653.45 which was consistent with the theoretical molecular weight of the protonated Dermaseptin-PP. Also, the intensity (%) of the impurities was under 10%, indicating the peptide Dermaseptin-PP was highly purified.

# Supplementary Tables

**Supplementary Table 1.** The settings of the RACE PCR program

| **Stage** | **Parameter** |
| --- | --- |
| Stage 1 | Initial denaturation at 94 ºC for 60 s |
| Stage 2 | 35 cycles (Denaturation at 94 ºC for 30 s; Primer annealing at 56 ºC for 30 s; Extension at 72 ºC for 180 s) |
| Stage 3 | Final extension at 72 ºC for 10 min |

**Supplementary Table 2.** Minimum inhibitory concentrations (MICs) and minimum bactericidal concentrations (MBCs) of Dermaseptin-PP against tested microbes.

| **Strains** | **MIC (**μM**)** | **MBC (**μM**)** |
| --- | --- | --- |
| *Escherichia coli* | 2 | 4 |
| *Staphylococcus aureus* | 2 | 2 |
| *Candida albicans* | 1 | 4 |
| *Enterococcus faecalis* | 4 | 4 |
| *Pseudomonas aeruginosa* | 2 | 4 |
| *Klebsiella Pneumoniae* | 1 | 2 |
| *MRSA* | 2 | 4 |
